# Editorial Chit Chat

**Published:** 1870-04

**Authors:** 


### EDITORIAL CHIT CHAT.

**An Anomaly.**

It is a little strange to us that M. L. Wood, Esq., of the Erie Road, should be so popular as a conductor, when any one with any knowledge of electricity whatever, knows full well, that Wood is a *non* conductor.

**Thanks.**

To Colonel E. L. Patrick for continued favors from State Head Quarters. And while we are at it, we desire to meddle in politics, just enough to say, that the Colonel is representing our county in the Legislature, ably and well, and that we would like to vote for his re-election.

**That Divorce.**

Mr. Jas. H. Mills, of this city, procured a divorce from his wife, Josephine, in the Indiana Courts recently. Mr. Mills could have procured a bill in this State as well, but the Laws of Indiana are such as to debar a divorced wife from recovering alimony, while it allows her free to marry at any time. Hence, the preference for an Indiana bill of divorce.

This little piece of information is entirely gratuitous—we publish it for the benefit of many who are sadly in need of it.

**"Poor Man With Large Family."**

We are frequently importuned for charity for a poor man with several small children. We always feel sorry for the children, but have very little sympathy for said "poor man." A poor man has no right to have more children than he can comfortably support, and we don't believe in paying a premium on a *poor* man's lust, any more than a *rich* ones. Poor preachers are often the subjects of much commiseration, in consequence of their large families. *We* have no sympathy for them—they are supposed to be intelligent men, or ought to be, therefore should know better than to give life to beings that they are unable to comfortably care for. We take no stock in poor but lustful preachers.

**An Old One.**

The Medical Record gives an account of what it claims to be the oldest relic of humanity on record. About two years ago a skeleton was deposited in the British Museum, which is said to belong to one of the earlier Pharaohs. It is encased in its original burial robes which are wonderfully perfect, considering their age. The lid of the coffin which contains the royal mummy is inscribed with the name of its occupant, "Pharaoh Mikerinus," who succeeded the heir of the builder of the Great Pyramids, about one thousand years before Christ. This royal mummy reigned in Egypt before Solomon was born, and is astonishing the Londoners with his majestic old carcass at this late day.

**Our Fire Department.**

We never saw a fire department like ours before. In most other cities, a man that "runs with the machine" is generally one of the b'hoys—is "on his muscle" and is posted with regard to the rules of the prize ring, and is always in "for a row" at a fire. Not so with *our* department—quite the contrary, we can assure you. Our fire companies are composed of our very best young men, and it is an honor to class them among your friends. In fact we have a "gentleman fire department," and a more efficient one does not exist anywhere.

Chief Engineer Sherman, has the honor of presiding over one of the most gentlemanly, best disciplined, and as thoroughly efficient fire department as exists in the State. Don't squint this way boys, this isn't a puff—it's a fact.

**"Baby Farming."**

An institution has been discovered in the goodly city of Philadelphia, where illegitimate infants are received, and so cared for, as to result in their death in a few months. A report made by the detectives to the Mayor of the city, reveals the fact that these little ones are received at this house by a woman, who states that she "makes it a business to take charge of new babies, and to *keep them until they die*." Her mode of feeding them, is to give the helpless little ones cow's milk, diluted with *three parts water*, boiled and sweetened with sugar. In this manner she has been in the habit of starving hundreds of innocent babes, and has received as compensation $3.00 a week for her damnable service, until the child dies.

Our students are in want of a subject for dissection—send the old hag up, we'll take her alive and cut her at our leisure.

**Medical.**

Dr. Earle, of Philadelphia, a Baptist Evangelist, extensively known as an eminent Physician, having practiced many years by mail, performing many remarkable cures, is now holding a series of religious meetings at Elimsport, in this county. While there he may be consulted in all difficult and chronic cures.—Williamsport *Gazette & Bulletin.*

We don't know the Reverend Doctor, but we'll wager a penny that he's an infernal knave. Any man that will use religion as an advertising medium by which to bring himself before the people as a *physician* is, in our opinion, a contemptable scoundrel, with religion worse than his physic.

**A "Shocking" Infant.**

There is a wonderful account in all the French papers of an astonishing infant that has just died, near Lyons, France. This infant was ten months old when it died, and was so charged with electricity that shocks were given to all persons in the same room with it. An intelligent body of physicians affirm, that at the moment of its death, its body became so luminous with electric light, as to dazzle the eyes of all present.

We have a pretty bright baby in our own household, who is continually *shocking* us with his odd pranks. We consider him quite as "wonderful" as the French baby. Doubtless, many of our readers can furnish similar ones.

**New Use for Old Things.**

Old shoes are gathered up in our cities, cut into small pieces, and placed for a few days in chloride of sulphur, which makes the particles of leather very hard and brittle. They are then washed, dried, ground to powder and mixed with shellac glue; it is then pressed into moulds where it is shaped into buttons, combs, knife-handles, &c.

The ladies of this city have found use for their old hoopskirts. They convert the steel springs therof into baskets, by bending them into any shape desired, and fastening them together with bits of wire, over which is pasted gilt paper. The baskets are then painted some bright color and are used as receptacles for fancy work, or are hung against the wall to receive papers, magazines, &c.

**That Fire Alarm.**

We heard it. The little bell in the 5th Ward awoke us, and by opening our chamber window and applying our hand to our ear, we were enabled to catch a faint sound from the monstrous bell perched upon the roof of the engine house. Gentlemen managers of the fire department, don't you know that there isn't a bell in the city beyond the size of a cow-bell that will not give a better alarm than your big, new bell? Don't you know that a bell placed in a deep well cannot be heard ringing twenty yards from the curb, while if hoisted a hundred feet above the earth might be heard as many miles? Boost up your bell—stick it sixty feet higher—remove it from the well of buildings that surrounds it, then, may be, us denizens of the 5th Ward may not have to rely upon the tinkling of our little engine bell, to arouse us in case of fire.

**A Skillful Surgeon Dead.**

Hazard Arnold Potter, M. D., a celebrated and skillful surgeon, who resided at Geneva, N. Y., died in that place a short time since. Dr. Potter had performed many critical surgical operations, and was regarded by the profession as a bold and skillful operator. He was the first surgeon who ever performed successfully, the operations for trephining the spine, and gastrotomy for the relief of intussusception of the bowels. He was among the first to perform ovariotomy, and had operated upon forty cases. He was the author of a new means of amputating at the hip joint, and had performed many other unique surgical operations, original with himself.

Dr. Potter was universally beloved for his goodness of heart, for responding promptly to the calls of the poor as well as the rich, when his professional services were demanded. He was a close student, a careful observer, a thoroughly educated physician, and had a great love for his profession. When *such* a man dies, community at large, have *indeed* met with a great loss.

**Mistaken Identity.**

We desire to relate a little incident which occurred in a family of one of our wealthiest citizens, nearly a year ago, but which is too good to be lost:—A gardner, while engaged about the premises of the gentleman referred to, discovered a small, fat, black animal, which was so very tame, and looked so cunningly from his small, black eyes, as to induce the man to pick him up in his arms. The more he caressed the little animal, the more he admired his beauty and docility, and at once concluded to carry it to the elegant parlors of his employer to be inspected by the household. The daughter, beautifully attired for a morning walk, took the little creature in her lap, fondled and petted it as woman only can, and dispatched the servant to the nearest saddlery establishment, for collar and chain with which to secure the new found treasure. In the meantime, many were the queries as to what *kind* of an animal it was. The father thought it was a coon, the hired man, a weazle, but the zoological descriptions of the animals mentioned did not correspond with the one in hand. So, a certain Alderman was sent for, who was known to be familiar with the animals of the country, and *his* opinion as to the identity of the little creature was demanded. Charley looked at it for a moment, and then, entrenching himself behind a large, easy chair, with fingers to his nose, and eyes twinkling with merriment, delivered himself of this oration:—" *Why, Doctor, that's the healthiest skunk I ever saw* !"

Tableau! during which, exit skunk on a long-handled shovel.

---

**Dr. Paul Schœppe.**

This physician, who had been sentenced to be hanged, in Carlisle, Pa., for the alleged poisoning of a maiden lady by the name of Steinecke, " is in a peck of trouble."

It will be remembered that the Governor respited him, at the solicitation of numerous physicians, who contended, and justly too, that the chemical evidence of poisoning, as presented at his trial, was not sufficiently satisfactory to warrant his condemnation.

In the meantime, a letter has been received by the Governor, from Professor Greist, of Berlin, Prussia, stating that a young man bearing precisely the same name and description, was convicted of grand larceny and forgery in Berlin, in 1862, and who afterward escaped to America. More singularly still, this young man's father was a minister, and also emigrated to America; and precisely the same facts exist with reference to Dr. Paul Schœppe, of Carlisle, Pa. Under this state of affairs, things began to look badly for the Doctor, until one Charles Pache, of New York, visited the Doctor in his cell, and testified that *this* Paul Schœppe is not the one against whom he was a witness for forgery, &c., in Berlin. Soon afterward, another friend of *our* Paul turns up—Mr. F. A. Botticher, also of New York, who states that he was a fellow student of Dr. Paul Schœppe, of Carlisle, in Zulichan, from 1859 to 1863, consequently, *our* Paul could not have been the villianous Paul who was tried in Berlin. The forger Paul, of Berlin, had his father mixed up in his affair. The alleged murderer Paul has his paternal ancestor in the same fix. The affair is rather a complicated one—hope the Governor will be able to unravel it in such a manner as to let *our* Paul go free.

---

**The Wizard Oilist,**

Who disported in this neighborhood lately, has a law suit pending at the Montgomery county court this week for pouring his stuff in a Canajoharie man's ear, whereby great injury was done to that organ. He had given $500 bail for a similar offense at the same place some time before. One of this party also got in trouble by enticing a young girl from home.—*Rondout Freeman.*

This is not the first instance of a ruined ear from pouring *benzine* in it. We have had two victims under our treatment for a very painful inflammation of the ear, caused by the use of the " wizard oil." People are for ever, buying such villianous stuff, and when they are repaid for it as above, we are more than half inclined to say—" served him right ! "

ASTHMA.—Persons who are sufferers from this distressing complaint, will be glad to learn of a process by which an attack can be arrested, or at least, greatly ameliorated. Procure some stramonium leaves, dry them in the sun, then immerse them in a solution of saltpetre—the solution to be made by placing, in a pint of water, all the saltpetre that the water will dissolve. Again dry the leaves, place a few in an old teacup and set them on fire. They will burn slowly, and will throw off much smoke, which must be inhaled. You will be surprised to see how quickly this inhalation will give relief. Have the leaves always ready to commence your inhalation, the moment you feel the symptoms of a threatened attack.

---

SMOKING.—We have never yet seen the man who was in any manner benefited by tobacco smoking, but we have been consulted by very many, who have been injured by it. And now, a new terror to smokers arises, when they are made familiar with the fact that they are rendering themselves constantly liable to syphilis, through smoking cigars.

A correspondent to the *Medical and Surgical Reporter*, writes that there is a young man engaged in making cigars in New York city, who is a victim of constitutional syphilis in an aggravated form. That "his lips and tongue are completely covered with mucous patches, a most offensive odor emanates from his whole body, especially from his breath, and a caries seems about attacking the bones of the nose."

This man is engaged daily, in making cigars. It is well known that in the making of a cigar, the leaf is moistened with saliva. How would our smoking friends enjoy a cigar of this person's manufacture? Bah! The thought is disgusting; yet, we doubt not, many persons have imbibed syphilitic poison in this manner, without ever being able to account for it.

